# One health approach on serosurvey of anti-*Leptospira* spp. in homeless persons and their dogs in South Brazil

**DOI:** 10.1016/j.onehlt.2022.100421

**Published:** 2022-07-25

**Authors:** Anahi Chechia do Couto, Mara Lucia Gravinatti, Maysa Pellizzaro, Louise Bach Kmetiuk, Ana Carolina Yamakawa, Evelyn Cristine da Silva, Laís Giuliani Felipetto, Hélio Langoni, André de Souza Leandro, Carlos Eduardo de Santi, Andrea Pires dos Santos, Alexander Welker Biondo

**Affiliations:** a Department of Veterinary Medicine, Federal University of Paraná, Curitiba, PR 80.035-050, Brazil; b Department of Preventive Veterinary Medicine and Animal Health, School of Veterinary Medicine, University of São Paulo, São Paulo, SP 05339-003, Brazil; c Department of Veterinary Hygiene and Public Health, School of Veterinary Medicine and Animals Science, São Paulo State University, Botucatu, SP 18618-000, Brazil; d Zoonoses Surveillance Unit, Municipal Secretary of Health, Foz do Iguaçu, PR 85869-675, Brazil; e College of Veterinary Medicine, Purdue University, West Lafayette, Indiana 47906, USA

**Keywords:** One health, Epidemiology, Social vulnerability

## Abstract

Although leptospirosis has been described as a worldwide bacterial zoonosis primarily affecting vulnerable populations, to date no study has focused on concomitant serosurvey of homeless persons and their dogs. The aim of the present study was, to use a One Health approach to serologically assess homeless persons and their dogs in 3 major cities of south Brazil (São Paulo, Curitiba, and Foz do Iguaçu). Environmental information was obtained with an epidemiological questionnaire given to all participants. A total of 200 human and 75 dog samples were tested for anti-*Leptospira* spp. antibodies to thirty different serovars using the microscopic agglutination test. None of the homeless persons were positive while 5 of the 75 (6.7%) dogs were positive. Among homeless population, 89% (177 of 200) were male, 61% (122 of 200) self-declared Non-white, and 67% (134 of 200) were educated up to the 8th school grade. Lower exposure of homeless persons to *Leptospira* spp. in the present study when compared to other vulnerable populations (slum and low-income residents) may be result of less direct exposure as they are able to rapidly change locations in response to flooding events. In addition, these results may reflect the effectiveness of a specific healthcare service provided to people living in the streets in the 3 cities. While dogs may be used as environmental sentinels for leptospirosis, the low seropositivity results found in this report may indicate low transmission risk to homeless owners in direct daily contact with their dogs.

## 1 Introduction

Leptospirosis, caused by a bacterium of genus *Leptospira*, is considered one of the neglected tropical diseases of public health importance worldwide [1]. Transmission may occur through contact with contaminated urine of sewer rats (*Rattus novergicus*) that serve as the main reservoir in urban settings, or through exposure to soil or water contaminated with *Leptospira* spp. [2]. This disease affects vulnerable populations in tropical countries due to socio-environmental issues including inadequate infrastructure and sanitation, water and soil contamination, garbage accumulation, and rodent proliferation; all of which are common scenarios in Brazilian slums [3,4].

Homeless populations worldwide live without adequate or permanent housing and have little access to public and private resources. These populations are susceptible to multimorbidity of infectious and mental illness, substance abuse, stigmatization, and interpersonal violence, and social exclusion [5]. In Brazil, the homeless population is estimated at 140,559 persons, primarily living in poor conditions of highly populated urban cities, along with 13,151 slums distributed in 734 cities of the 27 Brazilian states [6].

Dogs may share the same ecological bioaccumulation environment as owners, and may act as sentinels or reservoirs for several zoonotic diseases [7]. Among these, leptospirosis may be life-threatening to both humans and dogs as it can lead to multiple organ involvement and death in severe cases [8]. Although the World Health Organization (WHO) has defined neighborhood dogs as semi-dependent on multiple families for living maintenance [9]. The role of stray dogs and dogs owned by homeless persons in zoonotic diseases remains to be fully established, particularly in urban settings. Accordingly, the aim of this study was to serologically assess homeless persons and their dogs in São Paulo, Curitiba, and Foz do Iguaçu, 3 major cities of south Brazil.

## 2 Material and methods

### 2.1 Study area and sample collection

The study herein was conducted in 3 major cities of south Brazil. São Paulo (23°32′56″S; 46°38′20″W) is the largest Brazilian (and Latin American) city with 12.4 million inhabitants; Curitiba (25°25′42″S; 49°16′24″W) is the ninth largest Brazilian city with 1.8 million inhabitants; and Foz do Iguaçu (25°32′49″S; 54°35′18″W) is the largest Brazilian border city (adjacent to Paraguay and Argentina) with 258,000 inhabitants.

Homeless persons were contacted through local official health services and voluntarily participated by signed consent for themselves and their dogs. An epidemiological questionnaire was given to participants to assess environmental information while dogs were clinically examined. Municipality nurses collected blood from the people and certified veterinarians collected blood from the dogs. Serum samples were obtained by centrifugation, stored at −20 °C, and tested by microscopic agglutination test (MAT) to detect anti-*Leptospira* spp. antibodies, according to the Brazilian Ministry of Health [10] protocols.

A collection of 30 serovars stored at 28 °C in Ellinghausen McCullough-Johnson-Harris media was used, including Andamana, Australis, Autumnalis, Bratislava, Bataviae, Bovis, Canicola, Castellonis, Copenhageni, CTG, Cynopteri, Djasiman, Grippotyphosa, Guaricura, Hardjo, Hebdomadis, Icterohaemorraghiae, Javanica, Minis, Nupezo-01, Panama, Patoc, Pomona, Prajtino, Pyrogenes, Sentot, Shermani, Tarassovi Whitcombi, Wolffi. The 1:100 dilution was used as the cutoff point. If a sample was positive for more than one serovar, the highest titer was considered the causative infection. The 1:100 dilution was considered the cutoff point to determined exposure to Leptospira spp. as previous established [10]. If a sample was seropositive for more than one serovar, the highest titer was considered [11].

### 2.2 Statistical analysis

The data were tabulated, descriptive analysis was performed with the Epi Info version 7 statistical software (CDC, Atlanta, GA, USA), frequency distributions with 95% confidence intervals were calculated, and results were organized for presentation.

### 2.3 Ethical considerations

This research was approved by the Ethics Committee in Human Research at the Federal University of Paraná (Register 80,099,017.3.0000.0102 and Protocol 3.166.749), Ethics Committee in Animal Use at the Federal University of Paraná (Protocol 044/2016), Municipal Health Secretaries of Curitiba (Register 80,099,017.3.3002.0101 and Protocol 3.225.726) and São Paulo (Register 80,099,017.3.3004.0086 and Protocol 3,366,684). All were subordinated and approved by the National Human Ethics Research Committee of the Brazilian Ministry of Health. All research participants provided written informed consent.

## 3 Results

Of the 200 humans samples, there were 119 (59.5%) homeless persons sampled in São Paulo, 59 (29.5%) in Curitiba, and 22 (11.0%) in Foz do Iguaçu. No human samples were positive for the presence of anti-*Leptospira* spp. antibodies. While answers to the epidemiological questionnaire were obtained and gathered (Table 1), no associated risk factor was statistically significant due to the absence of seropositivity.

**Table 1:** Human epidemiological data.

1) Demographic profile		n	%
Curitiba		59	29.5
São Paulo		119	59.5
Foz do Iguaçu		22	11.0
	Total	200	100.0
Sex	Male	176	88.0
	Female	24	12.0
	Total	200	100.0
Race	White	54	27.0
(self-declaration)	Non-white	122	61.0
	Not rated	24	12.0
	Total	200	100.0
Age	<30 years old	42	21.0
	31–50 years old	81	40.5
	>50 years old	53	26.5
	Not rated	24	12.0
	Total	200	100.0
Educational	Up to 8th grade	134	67.0
Background	High School / University	40	20.0
	Not rated	26	13.0
	Total	200	100.0
City of origin	Local city	71	35.5
	Other cities	106	53.0
	Not rated	23	11.5
	Total	200	100.0
Travel to other cities	Yes	35	17.9
	No	128	64
	Not rated	37	18.5
	Total	200	100.0
2) Social profile			
Have seen rat			
	Yes	78	39.0
	No	79	39.5
	Not rated	43	21.5
	Total	200	100.0
Contact with family	Yes	91	45.5
	No	79	39.5
	Not rated	30	15.0
	Total	200	100.0
Resting place^a^	Street	89	44.5
	Shelter	31	15.5
	Hostel	106	53.0
	Not rated	19	9.5
	Total	245	
Pet owner	Yes	40	20.0
	No	130	65.0
	Not rated	30	15.0
	Total	200	100.0
Pet species^a^			
Dog owner		39	82.5
Cat owner		7	17.5
(Both)		6	15.0
	Total	40	100.0
Drug use	Yes	134	67.0
	No	39	19.5
	Not rated	27	13.5
	Total	200	100.0
Drug type^a^			
	Alcohol	85	42.5
	Tobacco	62	31.0
	Marijuana	53	26.5
	Cocaine	46	23.0
	Crack	35	17.5
	Other drugs	9	4.5
	Not rated	24	12.0
	Total	314	

a Multiple answer questions, with >200 responses. However, percentages were calculated to 200 persons.

Of the 75 dogs sampled, 41 (54.7%) were from São Paulo, 13 (17.3%) from Curitiba, and 21 (28.0%) from Foz do Iguaçu. All the dogs were adults of undefined breed. A total of 5 (6.7%) dogs tested positive for anti-*Leptospira* spp. with a low variable serological titer (Table 2). Four dog samples reacted to Icterohaemorraghiae serovar (titer 100); one co-reacted to Copenhageni (titer 100) and another to Pyrogenes (titer 200). The fifth positive sample co-reacted to Copenhageni (titer 1600) and Pyrogenes (titer 400). Results presented after exclusion of 35 of the 75 (46.7%) samples from dogs vaccinated against *Leptospira* spp.

**Table 2:** Distribution and frequency of presence of anti-*Leptospira* antibodies detected by IFA in dogs owned homeless persons in Curitiba, Foz do Iguaçu and São Paulo cities. Results after exclusion of 35/75 (46.67%) samples from vaccinated dogs for *Leptospira* spp.

City (State)	Dogs (n)	Titer	Frequency (%)
Curitiba -PR	13	0	–
Foz do Iguaçu -PR	21	0	–
São Paulo -SP	41^a^	0	–
		100	4/41 (9.7)
		200	2/41(4.8)
		400	1/41 (5.0)
		1600	1/41 (5.0)

a Three dogs showed reactivity for more than one *Leptospira* spp. serovar.

## 4 Discussion

To the authors knowledge, this study is the first epidemiological serosurvey to date of *Leptospira* spp. in homeless persons worldwide. Previous studies were case reports of *Leptospira* spp. infection in homeless or outdoor persons in London [12], Tokyo [13], Florida [14], Lisbon [15], and Marseille [16].

Although negative results for human leptospirosis in homeless persons of 3 major Brazilian cities was a pleasant surprise, human leptospirosis has sustained high endemic levels in Brazil, mostly in urban areas, with an average annual report of 3810 cases (1.9 cases/100,000), according to a recent 16-year (2000–2015) retrospective survey by the Brazilian Ministry of Health [17]. São Paulo State accounted for 11,884 cases, corresponding to 21% of the leptospirosis cases nationwide [17].

Furthermore, São Paulo City alone presented 2201 cases in a 10-year period (2007–2016) survey, mostly in males (82%), aged 20 to 59 years (65%), living in urban areas (86%), under flooding (39%) and in contact with rodents (36%) [18]. Interestingly, homeless persons in this study were also mostly males (88%), aged 31 to 50 years (41%), and reported contact with rats (39%). However, the westside region, where homeless in this study were sampled, has already presented the lowest overall leptospirosis frequency (7%) when compared to south (28%), east (24%) and north (19%) city regions [18]. Similarly, another study with 2000 interviews of homeless persons in São Paulo city have shown mostly men (86%), aged between 31 and 49 years (51%) [19], with over a half sleeping on streets instead shelters [20].

As a vulnerable and outdoor population, homeless people have greater environmental exposure to infectious diseases. Our research group has recently shown a 55% seropositive rate for COVID-19 among homeless persons in São Paulo, the highest prevalence worldwide at the time [21]. However, homeless people may be less directly exposed to leptospirosis, as they are able to rapidly change locations in response to flooding events. In comparison, residents of slums or low-income flooding areas who normally refuse to evacuate may be exposed to overflowing open sewers, which are 3-times more likely to contain pathogenic *Leptospira* spp. and have 6-times more pathogen load [22] compared to areas with closed sewers. Thus, as floods in tropical countries may directly increase *Leptospira* spp. infection and outbreaks [23], the lack of fixed housing may allow homeless persons to avoid infection by migrating within the city itself [24], and therefore decrease their exposure to the consequences of floods. Despite the study herein has not surveyed environmental Leptospira spp., a recent systemic review has shown that leptospiral organisms may grow into a biofilm in both nutrient-free and complex microbiota environments, remaining virulent for months, particularly in soils and sediments [25]. Thus, populations environmentally exposed to Leptospira spp., such as homeless persons and their animals, are likely highly exposed in endemic areas such as the three surveyed cities of São Paulo, Curitiba and Foz do Iguaçu.

Despite living outdoors, the homeless persons in this study had low (absent) environmental infection of *Leptospira* spp., similar to lower seropositivity for *Toxoplasma gondii* of other vulnerable Brazilian populations [26]. In that study, 36% of homeless persons were seropositive for *T. gondii*, compared to 57% of people in a riverside community and 80% of indigenous individuals [26]. While homeless persons in this study were less exposed to bacterial *Leptospira* spp. infection probably due to their ability to quickly relocation during flooding, their lower exposure to protozoan *T. gondii* infection was mostly attributed to habits of eating mainly processed food and lacking fresh vegetables and raw or undercooked meat in their daily diet. When considering vector-borne diseases, a previous study has indicated that homeless populations of Houston, Texas, USA may be at risk for Chagas disease, due to outdoors exposure to vectors including triatomines and blood-borne pathogen risk behaviors, such as drug use [27]. Further studies should be conducted to establish the occurrence of zoonotic pathogens among homeless persons, particularly those of environmental exposure.

The One Health approach herein has shown seropositive dogs with no concurrent human exposure, indicating that each pathogen may have different animal and environmental risks for causing human infection, and all should be surveyed together. In contrast, homeless persons are reportedly more exposed to diseases transmitted from human-to-human, such as sexually transmitted infections from unprotected sex and needle-sharing behavior (syphilis, HIV, and hepatitis [28], and COVID-19 [21].

Despite the lack of positive results for leptospirosis in homeless persons in this study, lack of adequate water and sanitation should be considered intrinsic human rights [29]. In addition, housing itself has been a social determinant of health [30] and health equity [31]. Negative human leptospirosis in this study may also be attributed to the Brazilian Unified Health System in the 3 cities operating the Street Clinic (healthcare for people living in the streets), which attempts to improve access to health services, a lack of which may substantially increase homeless persons vulnerability [32].

Besides the human leptospirosis cases, all 3 cities also had confirmed dog cases of leptospirosis [33]. However, the 7% frequency of positive dogs we found was lower than the 11% of street and shelter dogs in São Paulo [34], and 14% of dogs from a slum area in Curitiba, Parana State [34]. In a downtown urban area of Parana State, 2% of owners and 21% of dogs were serologically positive [35]. Although the frequency we found for dogs may have been lower due to a relatively high vaccination rate (47% of dogs), results were corroborated by absence of seropositive homeless persons. In addition to positivity, one dog presented high titers (400 and 1600) to two serovars within serogroup Icterohaemorragiae, the same as previous studies mentioned [35,36].

As limitations in the present study, human and dog leptospirosis were solely surveyed by serological methods, as association of serology and molecular tests may improve sensitivity and specificity of leptospirosis [37]. Nonetheless, a comparative study of MAT and PCR in acute leptospirosis found similar detection results [38].

As limitations in the present study, human and dog leptospirosis was surveyed only by serology with no molecular testing that might have increased the rate of detection. In addition, samples were obtained through contact with local health services, which may have missed new, non-registered and homeless persons refusing health assistance. The high percentage of vaccinated dogs may have indicated a biased sampling of well-treated dogs and a well-assisted homeless population. Finally, despite being a multicentric study, relatively low sampling per city may impair extrapolation of these results to other homeless populations in Brazil or worldwide. As a complex disease, leptospirosis has reportedly affected mostly vulnerable and low-income populations with several socio-environmental issues [39], which includes homeless populations. Thus, further studies should be conducted to confirm our results, including more sampling of homeless persons, other major cities in different regions, and different locations within each city.

In summary, lower exposure of homeless persons to *Leptospira* spp. in the present study when compared to other vulnerable populations (slum and low-income residents) may be the result of less direct exposure, as they are able to rapidly change locations in response to flooding events. In addition, such results may reflect the effectiveness of a specific healthcare service (Street Clinic) provided to people living in the streets in the 3 cities. While dogs may be used as environmental sentinels for leptospirosis, low seropositivity results in this study could indicate low transmission risk of direct daily contact with homeless owners.

## Funding

No funding.

## CRediT authorship contribution statement

**Anahi Chechia do Couto:** Data curation, Writing – original draft. **Mara Lucia Gravinatti:** Writing – original draft. **Maysa Pellizzaro:** Validation, Formal analysis, Writing – original draft. **Louise Bach Kmetiuk:** Writing – original draft, Writing – review & editing. **Ana Carolina Yamakawa:** Validation, Formal analysis, Writing – original draft. **Evelyn Cristine da Silva:** Validation, Formal analysis, Writing – original draft. **Laís Giuliani Felipetto:** Validation, Writing – original draft. **Hélio Langoni:** Validation, Formal analysis, Writing – original draft. **André de Souza Leandro:** Resources, Writing – original draft. **Carlos Eduardo de Santi:** Writing – original draft. **Andrea Pires dos Santos:** Writing – original draft, Writing – review & editing. **Alexander Welker Biondo:** Conceptualization, Methodology, Software, Validation, Investigation, Resources, Data curation, Writing – original draft, Writing – review & editing, Visualization, Supervision, Project administration, Funding acquisition.

## Declaration of Competing Interest

The authors declare that they have no known competing financial interests or personal relationships that could have appeared to influence the work reported in this paper.
